# Dietary Consumption of Antioxidant Vitamins in Relation to Prostate Cancer Risk in Japanese Men: The Japan Public Health Center-based Prospective Study

**DOI:** 10.2188/jea.JE20220235

**Published:** 2024-03-05

**Authors:** Sanyu Ge, Ling Zha, Tomotaka Sobue, Tetsuhisa Kitamura, Junko Ishihara, Motoki Iwasaki, Manami Inoue, Taiki Yamaji, Shoichiro Tsugane, Norie Sawada

**Affiliations:** 1 Environmental Medicine and Population Sciences, Department of Social Medicine, Osaka University Graduate School of Medicine, Osaka, Japan; 2 Department of Food and Life Science, Azabu University, Kanagawa, Japan; 3 Division of Epidemiology, National Cancer Center Institute for Cancer Control, Tokyo, Japan; 4 Division of Cohort Research, National Cancer Center Institute for Cancer Control, Tokyo, Japan; 5 Division of Prevention, National Cancer Center Institute for Cancer Control, Tokyo, Japan; 6 National Institute of Health and Nutrition, National Institutes of Biomedical Innovation, Health and Nutrition, Tokyo, Japan

**Keywords:** vitamin, prostate cancer, JPHC study, prospective cohort study, Japanese men

## Abstract

**Background:**

Many epidemiological studies have investigated dietary intake of antioxidant vitamins in relation to prostate cancer risk in Western countries, but the results are inconsistent. However, few studies have reported this relationship in Asian countries.

**Methods:**

We investigated the association between intake of vitamins, including lycopene, α-carotene, β-carotene, vitamin C, vitamin E, with prostate cancer risk in the Japan Public Health Center-based Prospective (JPHC) study. 40,720 men without history of cancer finished the food frequency questionnaire (FFQ) and were included in the study. Hazard ratios (HRs) and 95% confidence intervals (CIs) of prostate cancer risk were calculated according to the quintiles of energy-adjusted intake of vitamins using Cox models.

**Results:**

After an average of 15.2 years (617,599 person-years in total) of follow-up, 1,386 cases of prostate cancer were identified, including 944 localized cases and 340 advanced cases. No associations were observed in consumption of antioxidant vitamins, including α-carotene, β-carotene, vitamin C, and vitamin E, and prostate cancer risk. Although higher lycopene intake was associated with increased risk of prostate cancer (highest vs lowest quintile, HR 1.24; 95% CI, 1.04–1.47; *P* for trend = 0.01), there was a null association of lycopene intake with risk of prostate cancer detected by subjective symptoms (HR 1.12; 95% CI, 0.79–1.58; *P* for trend = 0.11).

**Conclusion:**

Our study suggested no association between antioxidant intake of vitamins and prostate cancer risk.

## INTRODUCTION

Prostate cancer is common in men, and the incidence is gradually increasing worldwide.^1^ According to the Global Cancer Observatory: CANCER TODAY (GLOBOCAN) project estimations in 2020, the prostate cancer incidence was 30.7 per 100,000 persons, being second only to lung cancer among men. In Japan, the incidence of prostate cancer increased from 30.4 per 100,000 in 2012 to 51.8 per 100,000 in 2020 and was even higher than that of lung cancer, with approximately 106,139 new cases in 2020.^1^^,^^2^ On the one hand, this increasing trend was related to the widespread use of prostate-specific antigen (PSA) screening as prostate cancer diagnosis.^3^ However, it is worth noting that population-based PSA screening was not recommended in recent years because of the problem of overdiagnosis.^4^

On the other hand, prior to the introduction of PSA screening, evidence of geographic variation and immigration epidemiology in prostate cancer incidence highlighted the potential role of lifestyle factors in disease risk.^5^^,^^6^ Previous studies examined the associations between lifestyle factors and prostate cancer risk. Older age, family history, and taller height were strongly associated with increased risk of prostate cancer.^3^ Increasing studies suggested the important role of diet in prostate cancer prevention.^7^ Vitamins, an essential component of dietary factors, played an important role in preventing cancer. Vitamins act as antioxidants to prevent damage to deoxyribonucleic acid (DNA) and cells.^7^ However, evidence of the protective effect of intake of vitamins for prostate cancer is controversial. The Health Professional Follow-Up Study (HPFS) found that lycopene intake was protective against prostate cancer,^8^^,^^9^ but other studies did not observe this association in the United States^10^^,^^11^ and the Netherlands.^12^ In addition, some review studies have suggested that evidence of lycopene in prostate cancer prevention is limited.^13^^,^^14^ However, most studies were from the United States, and the evidence on the association between lycopene intake and prostate cancer risk in the Asian population was deficient. Similarly, many studies have reported inconsistent results for α-carotene, β-carotene, vitamin C, and vitamin E.^10^^,^^12^^,^^15^^–^^19^ Although the protective effect observed in some studies might exist,^15^^,^^16^ three clinical trials on antioxidant vitamins supplementation and prostate cancer risk suggested no association.^20^^–^^22^ However, these clinical trials on vitamin supplementations were all in the United States. Many studies in western countries analyzed the association between dietary intake of vitamins and prostate cancer risk, but with inconsistent results. Despite differences in vitamin intake between eastern and western countries, only a few studies reported a association between vitamin intake and prostate cancer risk in Asia.^16^ Therefore, it is necessary to analyze the association between dietary intake of vitamins and prostate cancer risk in a large cohort among the Japanese population.

Herein, our study aimed to investigate the association of α-carotene, β-carotene, vitamin C, vitamin E, and lycopene with prostate cancer risk using the data from Japan Public Health Center-Based Prospective (JPHC) study. We also examined the effects of those vitamins on different stages of prostate cancer (localized and advanced cancer).

## METHODS

### Study population

The JPHC study had a total of 11 public health center regions, including Cohort I established in 1990 and Cohort II established in 1993. Participants in Cohort I ranged in age from 40 to 59 years and belonged to five Japanese Public Health Center regions (Iwate, Akita, Nagano, Okinawa Chubu, and Tokyo). In contrast, participants in Cohort II ranged in age from 40 to 69 years and belonged to six Japanese public health center regions (Ibaraki, Niigata, Kochi, Nagasaki, Okinawa-Miyako, and Osaka). Participants were informed of the purpose of the study and were mailed questionnaires at the beginning of the study, 5 years later, and 10 years later. In the current study, we chose the 5-year survey as the baseline because the second survey had more comprehensive nutritional data.

**Figure 1.**
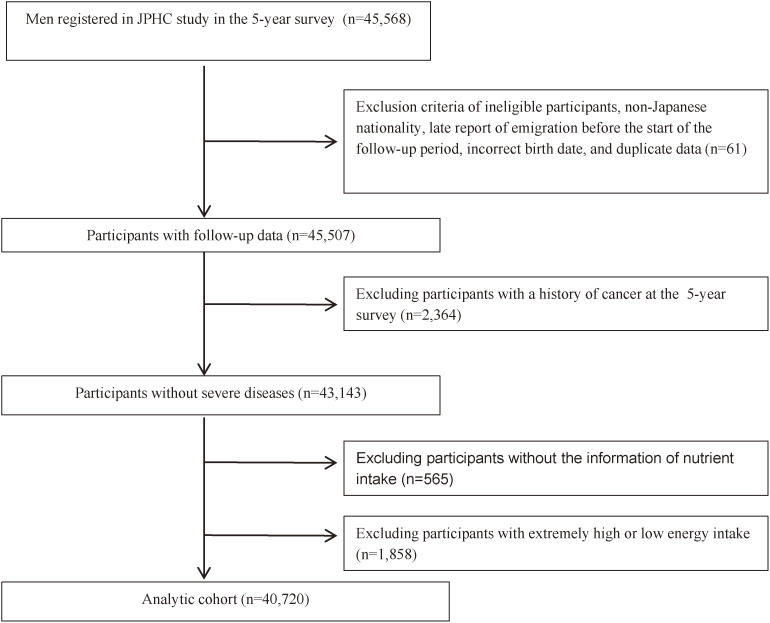
Participant flow chart

Participants from two public health centers (Tokyo and Osaka) were excluded because of lack of cancer incidence data in Tokyo Prefecture and different selection of participants in Osaka Prefecture. Among the eligible participants, 45,507 participants responded to the 5-year survey. We excluded participants with a history of cancer (*n* = 2,364) or who did not answer the food frequency questionnaire (FFQ) (*n* = 565). We also excluded 1,858 participants who reported excessive energy intake (<800 or >4,000 kcal). In total, we included 40,720 participants in this analysis (Figure 1). This study was approved by the Institutional Review Board of the National Cancer Center and National Cancer for Global Health and Medicine, Japan.

### Follow-up and identification of prostate cancer

The participants were followed from the start of the study (the 5-year follow-up survey) until the date of death, date of emigration from the study area, date of any cancer diagnosis, or the end of the study, whichever occurred first. The follow-up ended on December 31, 2015, in Iwate, Akita, Nagano, Okinawa Chubu, Ibaraki, Niigata, and Okinawa-Miyako; December 31, 2012, in Osaka; December 31, 2013, in Kochi; and December 31, 2014, in Nagasaki. We obtained follow-up-related information through the resident registry, and we treated those who were lost to follow up as censored data. Cancer-related data were obtained through local hospitals and cancer registries.

The third edition of the International Classification of Diseases for Oncology was used to classify prostate cancer. A total of 1,386 prostate cancer cases were newly diagnosed on December 31, 2015. We defined a cancer as advanced if it met any of the following criteria: 1) involvement of lymph nodes or other organs; 2) high Gleason score (8–10); or 3) poorly differentiated cases. If all the above information is missing, the status of prostate cancer is defined as undetermined. The remaining cases were localized prostate cancer. There were 340 advanced prostate cancer cases, 944 localized prostate cancer cases, and 102 cases of undetermined stages in this study.

### Dietary assessment

The FFQ in the 5-year survey was self-administered and obtained intake of 147 food and beverage items. Dietary consumption of the major antioxidant vitamins, namely α-carotene, and β-carotene, vitamin C, vitamin E (sum of α-, β-, γ-, and δ-tocopherols), and lycopene, were assessed at the 5-year follow-up survey. Daily nutrient intakes were then calculated with reference to the Standard Tables of Food Composition in Japan, and the amount of each antioxidant vitamin from each food item was estimated.^23^ In this study, vitamin supplements were not included in the exposure. All nutritional covariates were adjusted for total energy intake using the residual method.^24^

Validation for nutrition data in FFQ was evaluated using 14-day or 28-day dietary records, and reproducibility was evaluated using the 5-year follow-up questionnaire twice at approximately a 1-year interval.^25^ The Spearman correlation coefficients (energy-adjusted) for validity in men were as follows: 0.51 for cohort I and 0.47 for cohort II for α-carotene; 0.40 for cohort I and 0.46 for cohort II for β-carotene; 0.43 for cohort I and 0.48 for cohort II for vitamin C.^25^ For reproducibility, the coefficients were moderate: 0.46 for cohort II for lycopene; 0.48 for cohort I and 0.46 for cohort II for α-carotene; 0.43 for cohort I and 0.54 for cohort II for β-carotene; 0.67 for cohort I and 0.58 for cohort II for vitamin C.^25^^–^^27^

### Statistical analysis

In the study, we calculated quintile intakes of antioxidant vitamins and analyzed the distribution of nutrients and various possible risk factors for prostate cancer. We also analyzed the characteristics of prostate cancer according to different methods. Cox proportional hazards regression analysis was used to calculate hazard ratios (HRs) and 95% confidence intervals (CIs) of prostate cancer according to quintiles of dietary antioxidant intake of vitamins and adjusted for other confounding factors. We utilized Cox proportional hazards regression analysis to test for linear trends, which assigned median value in each quintile of vitamins intake.

The following factors were adjusted as confounding in the model: age; study area; smoking status (never, former, current, or missing), alcohol frequency (seldom, ≤4, ≥5 times/week, or missing), body mass index (<21, 21–22.9, 23–24.9, ≥25 kg/m^2^, or missing), physical activity (quintiles), history of diabetes mellitus (yes, no, or missing), past history of health check-up (any of the following: blood pressure, electrocardiogram, chest X-ray, gastric photofluorography, and fecal occult blood test; yes, no), green tea (quintiles), coffee (quintiles), energy-adjusted consumption of dairy products (quintiles) and soy food (quintiles). Because we did not collect information on PSA screening, we used a history of health check-up in the starting year of the study as a surrogate marker. Additionally, we conducted the same analysis according to the different stages of prostate cancer. To further rule out the influence of PSA screening, we limited the outcome to prostate cancer detected using subjective symptoms for sensitivity analysis. Two-sided *P* values <0.05 were regarded as statistically significant. The analyses were performed using Stata version 16.0 software (StataCorp, College Station, TX, USA).

## RESULTS

During 617,599 person-years of follow-up (mean, 15.2 years) in 40,720 men, a total of 1,386 prostate cancer cases were newly diagnosed and included in the analysis.

Table 1 shows the characteristics of the study participants according to the quintile of antioxidant vitamin intake. The men who consumed more lycopene underwent more health check-ups and had increased soy product intake. Participants who consumed more of other vitamins (carotenoids, vitamin C, and vitamin E) tended to be older, smoke more, drink less alcohol, and consumed more soy food and dairy products.

**Table 1.** Characteristics of study subjects according to quintile of antioxidant vitamins

	Men (*n* = 40,720)					

	Lowest	Second	Third	Fourth	Highest	*P* vaule^a^
**Lycopene**						
Number of participants, *n*	8,144	8,144	8,144	8,144	8,144	
Age, years, mean (SD)	57.4 (7.8)	57.1 (7.9)	58.0 (8.1)	57.0 (7.7)	57.1 (7.7)	<0.001
Body mass index, kg/m^2^, mean (SD)	23.6 (3.0)	23.5 (2.8)	23.5 (2.9)	23.7 (2.9)	23.7 (2.9)	<0.001
Physical activity, MET-h/d, mean (SD)	36.8 (10.5)	37.6 (10.2)	37.4 (10.2)	38.3 (10.2)	36.6 (9.9)	<0.001
Current smoker, %	4,001 (49.1%)	3,709 (45.5%)	3,271 (40.2%)	3,567 (43.8%)	3,289 (40.4%)	<0.001
Alcohol intake, ≥5 days/week, %	4,506 (55.3%)	3,945 (48.4%)	3,621 (44.5%)	4,261 (52.3%)	3,150 (38.7%)	<0.001
Past history of diabetes mellitus, yes, %	427 (5.2%)	444 (5.5%)	567 (7.0%)	511 (6.3%)	727 (8.9%)	<0.001
Health checkup, yes, %	6,143 (75.4%)	6,610 (81.2%)	6,830 (83.9%)	6,885 (84.5%)	6,961 (85.5%)	<0.001
Green tea intake, g/day, median (IQR)	300.0 (90.0–600.0)	300.0 (120.0–600.0)	325.7 (120.0–600.0)	325.7 (120.0–625.7)	300.0 (120.0–600.0)	<0.001
Coffee intake, g/day, median (IQR)	79.3 (0.0–256.4)	113.6 (25.7–290.7)	79.3 (0.0–245.0)	113.6 (25.7–275.7)	79.3 (25.7–196.4)	<0.001
**Dietary intake^b^**						
Total energy intake, kcal/day, median (IQR)	2,097.8 (1,662.5–2,582.1)	2,026.5 (1,634.5–2,476.0)	1,971.5 (1,600.7–2,425.2)	2,446.3 (2,115.4–2,831.9)	1,858.8 (1,588.3–2,120.7)	<0.001
Soy products intake, g/day, median (IQR)	60.3 (37.1–95.6)	65.9 (43.1–98.0)	69.1 (46.5–101.7)	72.3 (49.1–104.4)	78.6 (53.1–116.9)	<0.001
Dairy products intake, g/day, median (IQR)	65.1 (11.1–180.6)	89.0 (22.8–200.0)	109.7 (29.5–225.6)	107.0 (35.8–193.6)	127.1 (44.7–238.6)	<0.001
Lycopene, mcg/day, median (IQR)	91.9 (27.7–146.9)	339.1 (266.7–423.0)	834.2 (658.6–1,086.3)	2,529.5 (1,929.9–3,217.7)	6,587.9 (5,080.5–9,735.6)	<0.001

**α-carotene**						
Number of participants, *n*	8,144	8,144	8,144	8,144	8,144	
Age, years, mean (SD)	56.5 (7.9)	56.5 (7.7)	57.1 (7.7)	57.6 (7.7)	58.8 (8.0)	<0.001
Body mass index, kg/m^2^, mean (SD)	23.4 (2.9)	23.5 (2.8)	23.6 (2.9)	23.6 (2.8)	23.7 (2.9)	<0.001
Physical activity, MET-h/d, mean (SD)	36.6 (10.4)	37.5 (10.2)	37.7 (10.1)	37.8 (10.1)	37.2 (10.3)	<0.001
Current smoker, %	4,275 (52.5%)	3,904 (47.9%)	3,594 (44.1%)	3,308 (40.6%)	2,756 (33.8%)	<0.001
Alcohol intake, ≥5 days/week, %	4,637 (56.9%)	4,334 (53.2%)	3,941 (48.4%)	3,655 (44.9%)	2,916 (35.8%)	<0.001
Past history of diabetes mellitus, yes, %	444 (5.5%)	483 (5.9%)	531 (6.5%)	578 (7.1%)	640 (7.9%)	<0.001
Health checkup, yes, %	6,279 (77.1%)	6,742 (82.8%)	6,793 (83.4%)	6,860 (84.2%)	6,755 (82.9%)	<0.001
Green tea intake, g/day, median (IQR)	300.0 (90.0–600.0)	300.0 (120.0–600.0)	300.0 (120.0–600.0)	325.7 (120.0–625.7)	325.7 (120.0–625.7)	<0.001
Coffee intake, g/day, median (IQR)	113.6 (0.0–290.7)	113.6 (25.7–275.7)	113.6 (25.7–275.7)	94.3 (0.0–250.0)	79.3 (0.0–173.6)	<0.001
**Dietary intake^b^**						
Total energy intake, kcal/day, median (IQR)	2,030.9 (1,642.6–2,483.6)	2,210.2 (1,722.1–2,622.5)	1,993.7 (1,684.6–2,602.6)	2,174.5 (1,742.5–2,602.3)	1,936.3 (1,553.7–2,324.7)	<0.001
Soy products intake, g/day, median (IQR)	59.5 (36.4–91.1)	65.5 (42.8–96.1)	68.4 (45.9–100.0)	73.6 (49.5–108.0)	80.6 (53.6–120.1)	<0.001
Dairy products intake, g/day, median (IQR)	66.5 (15.3–182.1)	90.6 (26.8–195.4)	101.7 (29.9–212.1)	115.9 (36.3–213.8)	125.8 (34.5–241.8)	<0.001
α-carotene, mcg/day, mean (IQR)	82.4 (45.2–113.4)	221.6 (184.4–259.6)	390.1 (341.8–445.3)	654.9 (575.3–751.9)	1,261.1 (1,024.6–1,739.3)	<0.001

**β-carotene**						
Number of participants, *n*	8,144	8,144	8,144	8,144	8,144	
Age, years, mean (SD)	55.8 (7.7)	56.3 (7.7)	57.2 (7.7)	58.0 (7.8)	59.2 (7.9)	<0.001
Body mass index, kg/m^2^, mean (SD)	23.5 (3.0)	23.5 (2.8)	23.6 (2.8)	23.6 (2.9)	23.7 (2.9)	<0.001
Physical activity, MET-h/d, mean (SD)	36.7 (10.4)	37.6 (10.2)	37.7 (10.1)	37.5 (10.1)	37.2 (10.3)	<0.001
Current smoker, %	4,325 (53.1%)	3,981 (48.9%)	3,615 (44.4%)	3,192 (39.2%)	2,724 (33.4%)	<0.001
Alcohol intake, ≥5 days/week, %	4,786 (58.8%)	4,418 (54.2%)	3,949 (48.5%)	3,551 (43.6%)	2,779 (34.1%)	<0.001
Past history of diabetes mellitus, yes, %	417 (5.1%)	457 (5.6%)	532 (6.5%)	597 (7.3%)	673 (8.3%)	<0.001
Health checkup, yes, %	6,188 (76.0%)	6,663 (81.8%)	6,826 (83.8%)	6,917 (84.9%)	6,835 (83.9%)	<0.001
Green tea intake, g/day, median (IQR)	300.0 (60.0–600.0)	300.0 (120.0–600.0)	325.7 (120.0–600.0)	325.7 (145.7–625.7)	360.0 (145.7–625.7)	<0.001
Coffee intake, g/day, median (IQR)	120.0 (25.7–300.0)	120.0 (25.7–300.0)	113.6 (25.7–256.4)	79.3 (0.0–222.1)	60.0 (0.0–173.6)	<0.001
**Dietary intake^b^**						
Total energy intake, kcal/day, median (IQR)	2,047.1 (1,635.2–2,551.7)	2,120.0 (1,719.1–2,577.3)	2,129.6 (1,710.1–2,595.1)	2,091.2 (1,699.3–2,541.1)	1,938.2 (1,577.8–2,385.9)	<0.001
Soy products intake, g/day, median (IQR)	54.3 (32.6–84.8)	64.4 (42.6–94.4)	69.7 (47.9–100.6)	75.7 (51.3–110.2)	83.8 (56.7–122.7)	<0.001
Dairy products intake, g/day, median (IQR)	57.3 (13.7–173.0)	89.7 (26.3–196.7)	105.0 (32.7–204.4)	117.6 (36.0–219.9)	129.4 (38.8–244.5)	<0.001
β-carotene, mcg/day, mean (IQR)	873.8 (616.7–1,079.8)	1,605.5 (1,431.2–1,774.4)	2,329.9 (2,140.6–2,533.6)	3,292.9 (3,014.4–3,634.1)	5,326.6 (4,560.2–6,893.4)	<0.001

**Vitamin C**						
Number of participants, *n*	8,144	8,144	8,144	8,144	8,144	
Age, years, mean (SD)	55.1 (7.4)	56.0 (7.5)	57.1 (7.7)	58.3 (7.8)	60.0 (7.9)	<0.001
Body mass index, kg/m^2^, mean (SD)	23.7 (3.0)	23.6 (2.9)	23.5 (2.9)	23.6 (2.9)	23.6 (2.8)	0.088
Physical activity, MET-h/d, mean (SD)	36.9 (10.4)	37.5 (10.2)	37.5 (10.2)	37.5 (10.1)	37.3 (10.2)	<0.001
Current smoker, %	4,347 (53.4%)	3,959 (48.6%)	3,464 (42.5%)	3,262 (40.1%)	2,805 (34.4%)	<0.001
Alcohol intake, ≥5 days/week, %	5,002 (61.4%)	4,427 (54.4%)	3,979 (48.9%)	3,427 (42.1%)	2,648 (32.5%)	<0.001
Past history of diabetes mellitus, yes, %	424 (5.2%)	447 (5.5%)	539 (6.6%)	595 (7.3%)	671 (8.2%)	<0.001
Health checkup, yes, %	6,142 (75.4%)	6,635 (81.5%)	6,807 (83.6%)	6,882 (84.5%)	6,963 (85.5%)	<0.001
Green tea intake, g/day, median (IQR)	120.0 (25.7–300.0)	300.0 (120.0–420.0)	325.7 (145.7–600.0)	600.0 (300.0–720.0)	625.7 (300.0–1,320.0)	<0.001
Coffee intake, g/day, median (IQR)	120.0 (25.7–300.0)	120.0 (25.7–300.0)	94.3 (25.7–275.7)	79.3 (0.0–219.3)	60.0 (0.0–150.7)	<0.001
**Dietary intake^b^**						
Total energy intake, kcal/day, median (IQR)	2,055.1 (1,644.9–2,545.3)	2,102.5 (1,690.2–2,568.5)	2,099.5 (1,697.4–2,564.1)	2,073.4 (1,682.4–2,526.5)	1,996.3 (1,608.9–2,456.0)	<0.001
Soy products intake, g/day, median (IQR)	55.9 (33.3–87.6)	66.2 (43.6–98.3)	70.8 (47.7–104.6)	74.3 (50.9–108.5)	78.8 (53.7–115.4)	<0.001
Dairy products intake, g/day, median (IQR)	54.6 (12.2–166.4)	89.0 (25.0–195.8)	107.8 (32.3–211.7)	120.1 (38.5–225.8)	127.7 (40.3–234.3)	<0.001
Vitamin C, mg/day, mean (IQR)	46.6 (34.9–54.9)	75.4 (68.7–82.0)	102.1 (95.2–109.2)	135.2 (125.9–145.6)	197.3 (175.4–233.9)	<0.001

**Vitamin E**						
Number of participants, *n*	8,144	8,144	8,144	8,144	8,144	
Age, years, mean (SD)	56.2 (7.8)	56.5 (7.8)	57.2 (7.8)	57.6 (7.8)	59.0 (7.9)	<0.001
Body mass index, kg/m^2^, mean (SD)	23.4 (2.9)	23.5 (2.9)	23.5 (2.8)	23.6 (2.9)	23.8 (2.9)	<0.001
Physical activity, MET-h/d, mean (SD)	37.1 (10.5)	37.7 (10.3)	37.4 (10.1)	37.5 (10.1)	37.1 (10.1)	<0.001
Current smoker, %	2,148 (26.4%)	2,411 (29.6%)	2,724 (33.4%)	3,035 (37.3%)	3,419 (42.0%)	<0.001
Alcohol intake, ≥5 days/week, %	4,303 (52.8%)	3,912 (48.0%)	3,604 (44.3%)	3,255 (40.0%)	2,763 (33.9%)	<0.001
Past history of diabetes mellitus, yes, %	420 (5.2%)	428 (5.3%)	503 (6.2%)	562 (6.9%)	763 (9.4%)	<0.001
Health checkup, yes, %	6,223 (76.4%)	6,652 (81.7%)	6,837 (84.0%)	6,849 (84.1%)	6,868 (84.3%)	<0.001
Green tea intake, g/day, median (IQR)	300.0 (85.7–600.0)	300.0 (120.0–600.0)	325.7 (120.0–600.0)	325.7 (120.0–625.7)	325.7 (120.0–625.7)	<0.001
Coffee intake, g/day, median (IQR)	113.6 (0.0–300.0)	113.6 (25.7–300.0)	94.3 (25.7–250.0)	94.3 (25.7–250.0)	79.3 (0.0–173.6)	<0.001
**Dietary intake^b^**						
Total energy intake, kcal/day, median (IQR)	2,044.6 (1,638.0–2,489.6)	2,099.6 (1,695.5–2,552.0)	2,091.3 (1,690.1–2,562.8)	2,090.8 (1,699.0–2,562.5)	1,993.8 (1,597.2–2,494.3)	<0.001
Soy products intake, g/day, median (IQR)	42.6 (27.8–60.4)	59.5 (41.5–80.0)	70.5 (49.9–95.1)	84.1 (59.7–115.0)	116.7 (78.6–175.6)	<0.001
Dairy products intake, g/day, median (IQR)	65.1 (14.2–192.9)	91.2 (24.7–201.1)	107.1 (32.7–210.8)	112.0 (35.8–214.9)	119.3 (37.0–223.6)	<0.001
Vitamin E, mg/day, mean (IQR)	12.4 (10.5–13.7)	16.5 (15.7–17.3)	19.7 (18.9–20.4)	23.1 (22.1–24.2)	28.9 (26.9–32.4)	<0.001

BMI, body mass index; IQR, interquartile range; MET, metabolic equivalent; SD, standard deviation.

^a^Based on ANOVA or chi-square test.

^b^Intake was adjusted for energy intake with the residual method.

Table 2 shows the characteristics of prostate cancer diagnosed by different means. Of these, 305 cases were detected by competent symptoms, 480 by screening, 315 by accidental detection, and 286 by unclear methods. Of the prostate cancer diagnosed through screening, 79.1% were localized prostate cancer and 16.4% were advanced cases. However, 58.4% were localized prostate cancer and 38.7% were advanced cases detected by subjective symptoms.

**Table 2.** The characteristic of prostate cancer according to the method of detection

	Subjective symptoms	Screening	Incidentally	Unknown
Localized prostate cancer	178 (58.4%)	380 (79.1%)	238 (75.6%)	148 (51.7%)
Advanced prostate cancer	118 (38.7%)	79 (16.4%)	63 (20.0%)	80 (28.0%)
Undetermined	9 (2.9%)	21 (4.5%)	14 (4.4%)	58 (20.3%)

Total	305 (100%)	480 (100%)	315 (100%)	286 (100%)

Table 3 shows the multivariable-adjusted HRs and 95% CIs for prostate cancer risk according to the quintile of energy-adjusted intake of vitamins. Compared with the lowest group of lycopene intake, the HR for the highest group was 1.24 (95% CI, 1.04–1.47). We observed no association between other vitamins and prostate cancer risk (α-carotene: HR 1.03; 95% CI, 0.86–1.24, highest vs lowest quintile, *P* for trend = 0.56; β-carotene: HR 0.96; 95% CI, 0.80–1.16, *P* for trend = 0.39; vitamin C: HR 1.05; 95% CI, 0.86–1.30, *P* for trend = 0.81; and vitamin E: HR 1.00; 95% CI, 0.81–1.24, *P* for trend = 0.70). Considering that vitamins could influence prostate cancer progression, we examined localized and advanced prostate cancer separately. For localized cancer, there was a 33% risk increase in the highest lycopene intake (HR 1.33; 95% CI, 1.07–1.64, highest vs lowest quintile, *P* for trend = 0.03). However, there was a null association between lycopene intake and advanced prostate cancer risk (HR 1.00; 95% CI, 0.72–1.39, highest vs lowest quintile, *P* for trend = 0.36). No significant associations were found for other vitamins in advanced or localized prostate cancer risk.

**Table 3.** HRs and 95% CIs for prostate cancer according to quintile of energy-adjusted intake of lycopene, α-carotene, β-carotene, vitamin C and vitamin E

	Intake by quintile					
	Lowest	Second	Third	Fourth	Highest	*P* for trend
**Lycopene**						
Range, mcg/day	<204.4	204.4–525.0	525.1–1,445.2	1,445.3–4,029.7	>4,029.7	
**Total**						
Person-years	123,314	124,092	121,933	125,462	122,795	
Number of prostate cancer cases	256	247	301	274	308	
Model 1^a^	1.00	1.00 (0.84–1.19)	1.18 (1.00–1.39)	1.09 (0.92–1.30)	1.30 (1.10–1.54)	<0.001
Model 2^b^	1.00	0.97 (0.81–1.16)	1.12 (0.95–1.33)	1.05 (0.88–1.25)	1.24 (1.04–1.47)	0.01
**Localized**						
Person-years	123,314	124,092	121,933	125,462	122,795	
Number of prostate cancer cases	160	168	218	187	211	
Model 1^a^	1.00	1.09 (0.88–1.35)	1.39 (1.13–1.71)	1.20 (0.97–1.48)	1.43 (1.16–1.76)	<0.001
Model 2^b^	1.00	1.05 (0.84–1.30)	1.29 (1.05–1.58)	1.12 (0.90–1.39)	1.33 (1.07–1.64)	0.03
**Advanced**						
Person-years	123,314	124,092	121,933	125,462	122,795	
Number of prostate cancer cases	77	60	64	64	75	
Model 1^a^	1.00	0.80 (0.57–1.12)	0.81 (0.58–1.13)	0.84 (0.60–1.17)	1.01 (0.73–1.39)	0.36
Model 2^b^	1.00	0.79 (0.56–1.11)	0.81 (0.58–1.13)	0.82 (0.59–1.16)	1.00 (0.72–1.39)	0.36

**α-carotene**						
Range, mcg/day	<146.6	146.6–298.0	298.1–506.6	506.7–866.8	>866.8	
**Total**						
Person-years	122,736	124,207	124,252	123,723	122,677	
Number of prostate cancer cases	228	285	281	284	308	
Model 1^a^	1.00	1.22 (1.03–1.46)	1.16 (0.97–1.38)	1.11 (0.93–1.32)	1.10 (0.92–1.31)	0.97
Model 2^b^	1.00	1.18 (0.99–1.40)	1.10 (0.92–1.31)	1.05 (0.88–1.25)	1.03 (0.86–1.24)	0.56
**Localized**						
Person-years	122,736	124,207	124,252	123,723	122,677	
Number of prostate cancer cases	153	193	196	193	209	
Model 1^a^	1.00	1.23 (1.00–1.52)	1.20 (0.97–1.49)	1.12 (0.91–1.39)	1.11 (0.90–1.38)	0.99
Model 2^b^	1.00	1.16 (0.94–1.44)	1.12 (0.90–1.38)	1.03 (0.83–1.28)	1.01 (0.81–1.25)	0.47
**Advanced**						
Person-years	122,736	124,207	124,252	123,723	122,677	
Number of prostate cancer cases	60	80	65	65	70	
Model 1^a^	1.00	1.32 (0.94–1.84)	1.01 (0.71–1.44)	0.96 (0.68–1.37)	0.97 (0.68–1.38)	0.31
Model 2^b^	1.00	1.29 (0.92–1.81)	1.00 (0.70–1.43)	0.95 (0.66–1.36)	0.96 (0.67–1.38)	0.31

**β-carotene**						
Range, mcg/day	<1,256.4	1,256.4–1,952.1	1,952.2–2,762.1	2,762.2–4,039.9	>4,039.9	
**Total**						
Person-years	123,704	124,119	124,075	123,620	122,079	
Number of prostate cancer cases	214	252	311	320	289	
Model 1^a^	1.00	1.11 (0.92–1.33)	1.27 (1.07–1.51)	1.25 (1.05–1.48)	1.03 (0.86–1.23)	0.86
Model 2^b^	1.00	1.07 (0.89–1.28)	1.21 (1.01–1.45)	1.18 (0.99–1.41)	0.96 (0.80–1.16)	0.39
**Localized**						
Person-years	123,704	124,119	124,075	123,620	122,079	
Number of prostate cancer cases	135	178	209	229	193	
Model 1^a^	1.00	1.24 (0.99–1.55)	1.36 (1.09–1.69)	1.42 (1.15–1.76)	1.10 (0.88–1.37)	0.91
Model 2^b^	1.00	1.18 (0.94–1.48)	1.26 (1.01–1.57)	1.30 (1.05–1.63)	0.99 (0.78–1.24)	0.43
**Advanced**						
Person-years	123,704	124,119	124,075	123,620	122,079	
Number of prostate cancer cases	64	64	83	60	69	
Model 1^a^	1.00	0.94 (0.67–1.34)	1.13 (0.81–1.56)	0.78 (0.54–1.10)	0.84 (0.59–1.18)	0.15
Model 2^b^	1.00	0.92 (0.65–1.30)	1.11 (0.80–1.56)	0.76 (0.53–1.10)	0.81 (0.57–1.17)	0.14

**Vitamin C**						
Range, mg/day	<62.2	62.2–88.5	88.6–117.1	117.2–158.5	>158.5	
**Total**						
Person-years	125,015	125,291	124,283	123,714	119,293	
Number of prostate cancer cases	199	259	272	344	312	
Model 1^a^	1.00	1.18 (0.98–1.42)	1.14 (0.95–1.37)	1.32 (1.11–1.58)	1.12 (0.93–1.34)	0.33
Model 2^b^	1.00	1.14 (0.94–1.38)	1.09 (0.90–1.33)	1.25 (1.03–1.52)	1.05 (0.86–1.30)	0.81
**Localized**						
Person-years	125,015	125,291	124,283	123,714	119,293	
Number of prostate cancer cases	123	169	189	252	211	
Model 1^a^	1.00	1.25 (0.99–1.57)	1.30 (1.03–1.63)	1.59 (1.27–1.97)	1.26 (1.01–1.58)	0.05
Model 2^b^	1.00	1.18 (0.93–1.50)	1.20 (0.95–1.53)	1.45 (1.14–1.84)	1.12 (0.87–1.45)	0.57
**Advanced**						
Person-years	125,015	125,291	124,283	123,714	119,293	
Number of prostate cancer cases	63	71	68	65	73	
Model 1^a^	1.00	1.02 (0.73–1.44)	0.89 (0.63–1.25)	0.77 (0.54–1.09)	0.79 (0.56–1.12)	0.08
Model 2^b^	1.00	0.99 (0.70–1.40)	0.87 (0.60–1.25)	0.77 (0.52–1.13)	0.81 (0.54–1.21)	0.26

**Vitamin E**						
Range, mg/day	<14.7	14.7–18.1	18.2–21.3	21.4–25.4	>25.4	
**Total**						
Person-years	121,700	124,667	125,368	123,748	122,114	
Number of prostate cancer cases	240	252	290	296	308	
Model 1^a^	1.00	0.98 (0.82–1.17)	1.05 (0.89–1.25)	1.07 (0.90–1.28)	1.03 (0.87–1.23)	0.48
Model 2^b^	1.00	0.95 (0.79–1.14)	1.02 (0.84–1.22)	1.04 (0.86–1.27)	1.00 (0.81–1.24)	0.70
**Localized**						
Person-years	121,700	124,667	125,368	123,748	122,114	
Number of prostate cancer cases	160	180	201	201	202	
Model 1^a^	1.00	1.05 (0.85–1.29)	1.09 (0.88–1.34)	1.10 (0.89–1.35)	1.02 (0.82–1.26)	0.84
Model 2^b^	1.00	1.00 (0.80–1.25)	1.03 (0.82–1.29)	1.06 (0.84–1.34)	1.00 (0.77–1.29)	0.97
**Advanced**						
Person-years	121,700	124,667	125,368	123,748	122,114	
Number of prostate cancer cases	63	57	65	75	80	
Model 1^a^	1.00	0.84 (0.59–1.21)	0.89 (0.63–1.27)	1.02 (0.72–1.42)	1.02 (0.73–1.43)	0.52
Model 2^b^	1.00	0.83 (0.57–1.21)	0.89 (0.61–1.30)	0.97 (0.66–1.43)	0.93 (0.61–1.41)	0.86

CI, confidence interval; HR, hazard ratio.

^a^Models adjusted for age, study areas;

^b^Models adjusted for age, study areas, smoking, alcohol frequency, body mass index, physical activity, history of diabetes mellitus, health check-up and intake of green tea, coffee, dairy products, and soy.

Table 4 shows the multivariable-adjusted HRs and 95% CIs for risk of prostate cancer detected by subjective symptoms according to the quintile of energy-adjusted vitamins intake. Higher dietary vitamins intake was not associated with risk of prostate cancer. Compared with the lowest group of lycopene intake, the HRs and 95% CIs for lycopene were 1.12 (95% CI, 0.79–1.58) for total prostate cancer risk; 1.36 (95% CI, 0.85–2.19) for localized prostate cancer risk and 0.93 (95% CI, 0.54–1.59) for advanced prostate cancer risk, respectively.

**Table 4.** HRs and 95% CIs for prostate cancer detected by subjective symptoms according to quintile of energy-adjusted intake of lycopene, α-carotene, β-carotene, vitamin C and vitamin E

	Intake by quintile					
	Lowest	Second	Third	Fourth	Highest	*P* for trend
**Lycopene**						
Range, mcg/day	<204.4	204.4–525.0	525.1–1,445.2	1,445.3–4,029.7	>4,029.7	
**Total**						
Person-years	123,314	124,092	121,933	125,462	122,795	
Number of prostate cancer cases	65	49	65	53	73	
Model 1^a^	1.00	0.74 (0.51–1.07)	0.96 (0.68–1.35)	0.84 (0.59–1.21)	1.13 (0.81–1.59)	0.11
Model 2^b^	1.00	0.73 (0.50–1.06)	0.93 (0.65–1.32)	0.83 (0.57–1.20)	1.12 (0.79–1.58)	0.11
**Localized**						
Person-years	123,314	124,092	121,933	125,462	122,795	
Number of prostate cancer cases	32	27	41	34	44	
Model 1^a^	1.00	0.83 (0.50–1.39)	1.27 (0.80–2.02)	1.12 (0.69–1.82)	1.40 (0.88–2.21)	0.08
Model 2^b^	1.00	0.81 (0.48–1.37)	1.21 (0.75–1.94)	1.07 (0.65–1.75)	1.36 (0.85–2.19)	0.10
**Advanced**						
Person-years	123,314	124,092	121,933	125,462	122,795	
Number of prostate cancer cases	31	21	21	17	28	
Model 1^a^	1.00	0.66 (0.38–1.15)	0.62 (0.35–1.08)	0.55 (0.30–0.99)	0.89 (0.53–1.49)	0.64
Model 2^b^	1.00	0.67 (0.38–1.17)	0.63 (0.36–1.11)	0.57 (0.31–1.04)	0.93 (0.54–1.59)	0.49

**α-carotene**						
Range, mcg/day	<146.6	146.6–298.0	298.1–506.6	506.7–866.8	>866.8	
**Total**						
Person-years	122,736	124,207	124,252	123,723	122,677	
Number of prostate cancer cases	54	59	54	63	75	
Model 1^a^	1.00	1.05 (0.73–1.52)	0.88 (0.60–1.29)	0.96 (0.67–1.38)	0.94 (0.66–1.36)	0.72
Model 2^b^	1.00	1.03 (0.71–1.49)	0.85 (0.58–1.25)	0.94 (0.65–1.37)	0.93 (0.64–1.35)	0.87
**Localized**						
Person-years	122,736	124,207	124,252	123,723	122,677	
Number of prostate cancer cases	29	32	35	35	47	
Model 1^a^	1.00	1.05 (0.63–1.74)	1.03 (0.63–1.69)	0.95 (0.58–1.55)	0.99 (0.61–1.60)	0.86
Model 2^b^	1.00	1.00 (0.60–1.66)	0.98 (0.59–1.61)	0.90 (0.55–1.50)	0.98 (0.60–1.60)	0.97
**Advanced**						
Person-years	122,736	124,207	124,252	123,723	122,677	
Number of prostate cancer cases	24	25	19	24	26	
Model 1^a^	1.00	1.01 (0.58–1.78)	0.73 (0.40–1.33)	0.89 (0.50–1.57)	0.88 (0.50–1.56)	0.71
Model 2^b^	1.00	1.02 (0.58–1.80)	0.72 (0.39–1.33)	0.90 (0.50–1.61)	0.88 (0.49–1.58)	0.81

**β-carotene**						
Range, mcg/day	<1,256.4	1,256.4–1,952.1	1,952.2–2,762.1	2,762.2–4,039.9	>4,039.9	
**Total**						
Person-years	123,704	124,119	124,075	123,620	122,079	
Number of prostate cancer cases	56	50	64	69	66	
Model 1^a^	1.00	0.82 (0.56–1.20)	0.96 (0.67–1.38)	0.96 (0.67–1.36)	0.76 (0.53–1.10)	0.23
Model 2^b^	1.00	0.81 (0.55–1.18)	0.94 (0.65–1.35)	0.95 (0.66–1.36)	0.75 (0.51–1.09)	0.32
**Localized**						
Person-years	123,704	124,119	124,075	123,620	122,079	
Number of prostate cancer cases	27	29	38	45	39	
Model 1^a^	1.00	0.99 (0.58–1.67)	1.17 (0.72–1.92)	1.26 (0.78–2.04)	0.87 (0.53–1.43)	0.54
Model 2^b^	1.00	0.97 (0.57–1.65)	1.15 (0.69–1.90)	1.26 (0.77–2.07)	0.88 (0.52–1.48)	0.73
**Advanced**						
Person-years	123,704	124,119	124,075	123,620	122,079	
Number of prostate cancer cases	26	21	25	21	25	
Model 1^a^	1.00	0.75 (0.42–1.33)	0.82 (0.47–1.41)	0.65 (0.36–1.15)	0.71 (0.40–1.23)	0.27
Model 2^b^	1.00	0.74 (0.41–1.32)	0.82 (0.46–1.43)	0.64 (0.35–1.15)	0.69 (0.38–1.24)	0.33

**Vitamin C**						
Range, mg/day	<62.2	62.2–88.5	88.6–117.1	117.2–158.5	>158.5	
**Total**						
Person-years	125,015	125,291	124,283	123,714	119,293	
Number of prostate cancer cases	54	54	56	72	69	
Model 1^a^	1.00	0.90 (0.62–1.31)	0.83 (0.57–1.21)	0.96 (0.67–1.37)	0.83 (0.58–1.19)	0.45
Model 2^b^	1.00	0.89 (0.60–1.31)	0.83 (0.56–1.23)	0.97 (0.66–1.43)	0.87 (0.57–1.32)	0.92
**Localized**						
Person-years	125,015	125,291	124,283	123,714	119,293	
Number of prostate cancer cases	29	25	35	48	41	
Model 1^a^	1.00	0.78 (0.46–1.33)	0.98 (0.60–1.61)	1.20 (0.76–1.92)	0.93 (0.57–1.51)	0.79
Model 2^b^	1.00	0.79 (0.46–1.36)	1.01 (0.60–1.70)	1.29 (0.77–2.14)	1.03 (0.60–1.79)	0.50
**Advanced**						
Person-years	125,015	125,291	124,283	123,714	119,293	
Number of prostate cancer cases	23	28	20	23	24	
Model 1^a^	1.00	1.08 (0.62–1.88)	0.68 (0.37–1.24)	0.70 (0.39–1.26)	0.65 (0.36–1.17)	0.07
Model 2^b^	1.00	1.05 (0.59–1.84)	0.66 (0.35–1.25)	0.69 (0.36–1.30)	0.67 (0.34–1.32)	0.30

**Vitamin E**						
Range, mg/day	<14.7	14.7–18.1	18.2–21.3	21.4–25.4	>25.4	
**Total**						
Person-years	121,700	124,667	125,368	123,748	122,114	
Number of prostate cancer cases	56	45	70	66	68	
Model 1^a^	1.00	0.77 (0.52–1.15)	1.08 (0.76–1.54)	0.99 (0.69–1.42)	0.85 (0.59–1.22)	0.65
Model 2^b^	1.00	0.76 (0.51–1.14)	1.10 (0.75–1.61)	1.02 (0.68–1.54)	0.88 (0.56–1.36)	0.99
**Localized**						
Person-years	121,700	124,667	125,368	123,748	122,114	
Number of prostate cancer cases	31	30	47	36	34	
Model 1^a^	1.00	0.93 (0.56–1.54)	1.27 (0.81–2.01)	0.94 (0.58–1.53)	0.69 (0.42–1.14)	0.11
Model 2^b^	1.00	0.91 (0.54–1.53)	1.28 (0.78–2.10)	0.98 (0.57–1.69)	0.76 (0.42–1.38)	0.45
**Advanced**						
Person-years	121,700	124,667	125,368	123,748	122,114	
Number of prostate cancer cases	23	15	20	27	33	
Model 1^a^	1.00	0.63 (0.33–1.20)	0.78 (0.42–1.42)	1.04 (0.59–1.82)	1.17 (0.67–2.02)	0.19
Model 2^b^	1.00	0.62 (0.32–1.22)	0.80 (0.42–1.53)	1.04 (0.55–1.98)	1.11 (0.56–2.20)	0.27

CI, confidence interval; HR, hazard ratio.

^a^Models adjusted for age, study areas;

^b^Models adjusted for age, study areas, smoking, alcohol frequency, body mass index, physical activity, history of diabetes mellitus, health check-up and intake of green tea, coffee, dairy products, and soy.

eTable 1 shows antioxidant vitamins intake characteristics among the people who underwent health check-ups and those who did not. The lycopene intake of people who participated in health check-up (median 911.7; interquartile range [IQR], 290.9–3,360.2 mcg/day) was higher than that of people who did not (median 561.5; IQR, 177.2–2,493.2 mcg/day). However, there was little difference between people who took or did not take other vitamins.

## DISCUSSION

The present large prospective cohort study involving 40,720 participants and 1,386 cases investigated the association between antioxidant vitamins and prostate cancer risk. The results showed that intakes of α-carotene, β-carotene, vitamin C, and vitamin E were not significantly associated with risk of prostate cancer. Lycopene intake was associated with an increased risk of prostate cancer, especially for localized prostate cancer. However, there was no association between lycopene intake and advanced prostate cancer risk. The consumption of vitamins was not associated with various stages of risk of prostate cancer detected by subjective symptoms.

With the popularity of PSA screening, prostate cancer is susceptible to over diagnosis.^28^ In recent years, the 5-year survival rate of localized prostate cancer in Japan was 100%. These low-grade prostate cancers, such as those with Gleason score lower than 6, are responsible for the majority of detected cancer outcomes, but are not similarly associated with cancer progression.^29^ Also, the act of screening was associated with health consciousness.^30^ Participants with greater health consciousness who consumed more lycopene could be more likely to undergo screening, which might lead to an increased diagnosis of prostate cancer, particularly localized cancer, thereby leading to this seemingly positive association between lycopene and prostate cancer risk.

In order to reduce this bias, we also performed an analysis of prostate cancer detected by subjective symptoms. Our study found there was a null association between lycopene intake and risk of prostate cancer detected by subjective symptoms. Findings from prospective studies are inconsistent with regard to lycopene intake. Follow-up studies conducted by health professionals found that lycopene intake was associated with a decreased risk of prostate cancer, including lethal prostate cancer.^8^^,^^9^ Three studies have suggested a null association between dietary lycopene intake and prostate cancer risk.^11^^,^^12^^,^^15^ In serum lycopene levels, two studies suggested a protective effect for prostate cancer,^31^^,^^32^ and three studies supported no association.^33^^–^^35^ The seemingly positive association between localized prostate cancer risk and lycopene intake could be explained by confounding related to PSA screening. In a previous JPHC study, there was no association between fruit and vegetable intake and risk of prostate cancer.^36^ Therefore, it is plausible that lycopene intake was not associated with risk of advanced prostate cancer and prostate cancer detected by subjective symptoms.

As an antioxidant, α-carotene and β-carotene can be converted into vitamin A in the human body, preventing prostate cancer.^37^ However, our study showed that α-carotene and β-carotene were not associated with the risk of prostate cancer or prostate cancer detected by subjective symptoms, consistent with the Netherlands cohort study^12^ and The Prostate, Lung, Colorectal and Ovarian (PLCO) trials.^10^ Another Japanese study showed that α-carotene was related to reduce the risk of prostate cancer, whereas β-carotene was not.^16^ Two meta-analyses showed different results; whereas one showed a null association,^19^ the other showed that only α-carotene was associated with lowering the risk of prostate cancer but not advanced prostate cancer.^38^

There have been many studies on the association of vitamin C and vitamin E intake with prostate cancer risk. We did not observe an association between vitamin C and prostate cancer risk, which agrees with recent findings from several cohort studies of diet^10^^,^^12^^,^^15^ and two clinical trials of vitamin C supplementation.^20^^,^^22^ We also found that vitamin E was not associated with prostate cancer risk. Recent clinical trials showed that vitamin E did not decrease prostate cancer risk in the Physicians’ Health Study II Randomized Controlled Trial and Selenium and Vitamin E Cancer Prevention Trial.^20^^,^^21^ Similarly, there was a null association between vitamin E and prostate cancer risk in the Netherlands and Danish cohort study.^12^^,^^15^

There was little difference in dietary intake for vitamins other than lycopene between people who underwent health check-ups and those who did not (eTable 1). Therefore, the difference in these nutrients might also be small in people receiving PSA screening. Consequently, this detection bias caused by PSA screening confounding was more significant in lycopene than in other vitamins. There are some possible explanations for this phenomenon. In Japan, the sources of lycopene are limited, mainly tomatoes, persimmons, watermelons, and related tomato products. Other vitamins have a broader food source than lycopene, and are obtained from fruits and vegetables and dairy products, meat, and fish.^23^^,^^39^^,^^40^ However, due to the limited sources of lycopene, lycopene intake may vary among people who eat or do not consume tomato products.

### Strengths and limitations

Our study had several strengths. Our study involved 40,720 participants and 1,386 cases. We further investigated the effects of vitamins on different stages of prostate cancer. Information about vitamins was collected before outcomes appeared and could avoid recall bias. Our study has several limitations. First, we used only the 5-year FFQ at a single point. There could be some misclassifications due to changes of vitamins intake during the study period. If present, however, such misclassification would probably be non-differential and underestimate the true associations,^41^ Second, we were unable to consider the effects of PSA screening on the association between vitamin intake and prostate cancer because we did not collect information on PSA screening among all subjects. However, we analyzed to limit cases with subjective symptom to minimize the effects of PSA screening on prostate cancer. Additionally, we adjusted a history of health check-up as a surrogate marker for PSA screening, because people who receive a health check-up would be likely to have received the PSA screening. Finally, although we adjusted the multivariable model, unmeasured variables and residual confounding could still exist.

### Conclusion

In conclusion, we found that intakes of α-carotene, β-carotene, vitamin C, and vitamin E were not associated with prostate cancer risk. Lycopene intake was positively associated with risk of localized prostate cancer, which might be caused by PSA screening confounding. However, no association was observed between lycopene intake and risk of advanced prostate cancer and prostate cancer detected by subjective symptoms.

## SUPPLEMENTARY MATERIAL

eTable 1. Characteristics of antioxidant vitamins intake among the people who underwent health check-up or not
