# Supplementary material for: Dietary Consumption of Antioxidant Vitamins in Relation to Prostate Cancer Risk in Japanese Men: The Japan Public Health Center-based Prospective Study
Source: J Epidemiol. 2024 Mar 5;34(3):144–53. doi: 10.2188/jea.JE20220235 (PMC10853041; doi:10.2188/jea.JE20220235)
Supplement: Supplementary file 1 [file je-34-144-s001.pdf]

eTable 1. Characteristics of antioxidant vitamins intake among the people who underwent health check-up or not

	subject who didn't undergo health check-up	subject who underwent health check-up
Number	7,291	33,429
lycopene, mcg/day, median (IQR)	561.5 (177.2–2,493.2)	911.7 (290.9–3,360.2)
α-carotene, mcg/day, median (IQR)	351.3 (143.7–714.5)	400.6 (193.2–758.4)
β-carotene, mcg/day, median (IQR)	2,076.0 (1,195.5–3,387.4)	2,381.0 (1,482.5–3,680.6)
Vitamin C, mg/day, median (IQR)	91.1 (58.8–134.8)	104.2 (71.1–148.0)
Vitamin E, mg/day, median (IQR)	18.7 (14.5–23.4)	19.9 (15.9–24.3)

IQR, interquartile range.
